# The Role of Four-Dimensional Automatic Right Ventricular Quantification Technology to Determine RV Function and Hemodynamics in Patients With Pulmonary Hypertension Compared With Right Heart Catheterization

**DOI:** 10.3389/fcvm.2021.628610

**Published:** 2021-07-14

**Authors:** Weichun Wu, Bingyang Liu, Min Huang, David H. Hsi, LiLi Niu, Yue Tian, Jingru Lin, Jiangtao Wang, Shuai Yang, Hongquan Lu, Changming Xiong, Zhenhui Zhu, Hao Wang

**Affiliations:** ^1^State Key Laboratory of Cardiovascular Disease, Department of Echocardiography, National Center for Cardiovascular Diseases, Fuwai Hospital, Chinese Academy of Medical Sciences and Peking Union Medical College, Beijing, China; ^2^State Key Laboratory of Cardiovascular Disease, Department of Cardiology, Pulmonary Vascular Disease Center, National Center for Cardiovascular Diseases, Fuwai Hospital, Chinese Academy of Medical Sciences and Peking Union Medical College, Beijing, China; ^3^Department of Ultrasound, Meishan People's Hospital, Meishan, China; ^4^Heart and Vascular Institute, Stamford Hospital, Stamford, CT, United States; ^5^General Electric Healthcare, Beijing, China; ^6^Department of Cardiology, Capital Institute of Pediatrics, Beijing, China

**Keywords:** pulmonary hypertension, 4D-echocardiography, strain, right heart catheterization, right heart function

## Abstract

**Background:** Four-dimensional automatic right ventricular quantification technology (4D auto-RVQ) is a new method that can simultaneously measure right ventricular (RV) structure and strain. The role of 4D auto-RVQ in determining RV function and hemodynamics is not clear. The role of 4D auto-RVQ in determining RV function and hemodynamics is not clear. We assessed the 4D auto-RVQ to measure right heart structure, function, and hemodynamics in patients with pulmonary hypertension (PHTN) correlated with right heart catheterization (RHC).

**Methods:** We enrolled a prospective cohort of 103 patients with PHTN and 25 healthy controls between September 2017 and December 2018. All patients with PHTN underwent echocardiography and RHC. Patients were included if they underwent two-dimensional (2D) and 4D auto-RVQ echocardiographic sequences on the same day as RHC. We analyzed RV functional indices using 2D and 4D auto-RVQ analyses. We divided patients with PHTN into three groups according to echocardiographic image quality as follows: high (*n* = 24), average (*n* = 48), and poor (*n* = 4). Hemodynamic parameters were measured using RHC, including mean right atrial pressure, mean pulmonary arterial pressure, RV cardiac index (RV-CI), and pulmonary vascular resistance.

**Results:** There were significant differences in most 2D and 4D auto-RVQ parameters between patients with PHTN and healthy controls. Interobserver variability showed significant agreement with 4D auto-RVQ for most measurements except for 4D end-diastolic volume. Indices measured by auto 4D-RVQ in the high-quality image group had a good correlation with RHC but not in the average- and poor-quality image group. Mid-RV diameter showed the best predictive power for the right RV-CI [area under the curve (AUC) 0.935; 95% confidence interval (CI), 0.714–0.997; *p* < 0.001]. RV end-systolic volume >121.50 mL had a 71.43% sensitivity and a 100% specificity to predict right RV-CI (AUC, 0.890; 95% CI, 0.654–0.986; *p* < 0.001).

**Conclusions:** 4D auto-RVQ may be used to estimate RV function and some hemodynamic changes compared with RHC in PHTN patients with high image quality. Furthermore, a large sample of the study is needed to evaluate RV function by 4D auto-RVQ in PHTN patients with average image quality.

## Introduction

Echocardiography is the most commonly used imaging technique for the study of right ventricular (RV) morphology, volume, function, and tissue characterization (1, 2). The accuracy of 2DE is inferior to cardiac magnetic resonance imaging (3). Because of the complex RV geometry, 2DE cannot capture RV inflow and outflow in the same image acquisition. Real-time three-dimensional echocardiography (3DE), also named four-dimensional echocardiography (4DE), is a more accurate and quicker method to assess RV volume and function than 2DE, but in some cases, this approach still poses some technical difficulties (4).

The right ventricle has a unique crescent shape and complex muscle moment, which influences the accurate evaluation of RV function. In patients with pulmonary hypertension (PHTN), the right heart chambers are significantly enlarged, and the scanning width of the echocardiography is insufficient to cover the entire right ventricle. The walls of the right ventricle can be difficult to identify. RV strain has been extensively used to evaluate the myocardial function and has predictive potential in patients with PHTN. RV strain is also recommended in European guidelines as part of the echocardiographic assessment of the right heart in adults (5, 6).

Right heart catheterization (RHC) is recommended to confirm the diagnosis of pulmonary arterial hypertension (PAH) and to support treatment decisions (7). RV hemodynamic deterioration, which can be measured by RHC, such as changes in mean pulmonary arterial pressure (PAP), pulmonary vascular resistance (PVR), and decrease in RV cardiac index (CI), is associated with poor clinical outcomes in patients with PHTN (8). PHTN is a progressive and life-threatening disease leading to RV pressure overload, dysfunction, and ultimately death. RHC can reliably measure indices of right heart function, such as cardiac output (CO) and RV-CI.

Recently, 4D automatic RV quantification (4D auto-RVQ) has emerged as a new technology for comprehensive RV assessment. This technique can simultaneously measure RV volume, tricuspid annular plane systolic exertion (TAPSE), RV diameter, 4D ejection fraction (4D-EF), and longitudinal strain of the RV free wall and septum (9). In our study, we attempted to use this technology to estimate right heart function and important hemodynamic indices, as well as to obtain other indirect information on right heart structure and function in patients with PHTN.

## Materials and Methods

### Study Population

Our study was a prospective cross-sectional project by design. Adult inpatients with PHTN and healthy volunteers were enrolled from September 2017 to December 2018. All patients with PHTN underwent echocardiography and RHC. Patients with PHTN were included if they had a diagnosis of idiopathic PAH, chronic thromboembolic PAH, connective tissue disease PAH, or residual PAH after surgery for congenital heart disease by RHC with a mean PAP of >25 mm Hg (7). Patients were included if they underwent 2D and 4D auto-RVQ on the same day. Patients were excluded if they were <18 years of age, if echocardiographic images were inadequate for tracing. The exclusion criteria included congenital heart disease before the operation, serious valvular heart disease, significant coronary heart disease, atrial fibrillation, acute heart failure, renal or hepatic failure, and chronic obstructive pulmonary disease. Healthy adult volunteers served as healthy controls. These volunteers had no cardiac defects or family history of cardiac disease.

Our primary endpoint is the role of 4D auto-RVQ to determine RV function and hemodynamics in PHTN patients compared with RHC data. The secondary endpoint is the accuracy and characteristic of 4D auto-RVQ indices in PHTN patients compared to normal people.

Written informed consent was obtained from all participants or their legal representatives. The present study was approved by the ethics committee of Fuwai Hospital (no. 2018-1063). All procedures were performed in accordance with the 1964 Helsinki Declaration and its later amendments.

### 2DE Acquisition and Analysis

All patients underwent standard transthoracic echocardiography using a GE Vivid E9 (GE Healthcare, Milwaukee, WI, USA) with a 3.5-MHz phased-array transducer. Consecutive cardiac cycles were recorded during breath-holding with stable electrocardiography tracing. All patients underwent standard 2DE and Doppler echocardiography examinations with detailed evaluation of right heart function. A comprehensive evaluation of the right ventricle by 2DE obtained six standardized views, including the parasternal long-axis, RV inflow, parasternal short-axis, apical four-chamber, and subcostal views.

Offline analysis was then performed on digitally stored images. Indices of 2DE included TAPSE and RV fractional area change. Right heart size was quantified as RV end-diastolic area at the end of the electrocardiogram (ECG) T-wave. RV chamber size was assessed at the apex of the ECG R-wave in the apical four-chamber view with a focus on the right ventricle. We measured pulmonary systolic pressure by Doppler tricuspid regurgitation peak velocity plus estimated right atrial pressure.

### 4D Auto-RVQ Acquisition and Analysis

**Figure 1:**
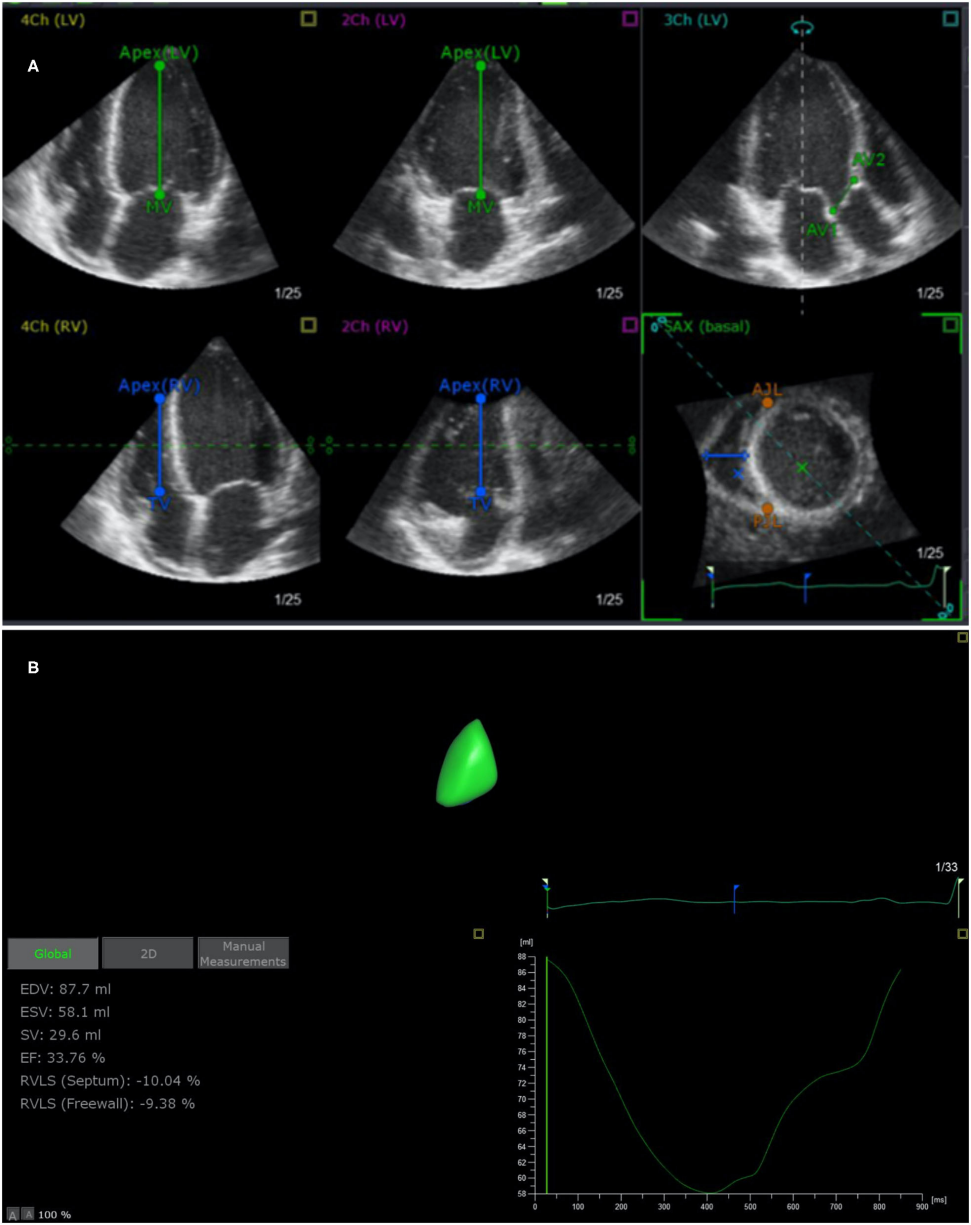
End-systolic verification and editing of endocardial RV borders using TomTec 4D RV Function 2.0 **(A)** and 4D auto-RVQ measurement **(B)**. RV, right ventricular; EDV, end-diastolic volume; ESV, end-systolic volume; EF, ejection fraction; RVLS (septum), right ventricular longitudinal strain of the septum; RVLS (free wall), right ventricular longitudinal strain of the free wall; TAPSE, tricuspid annular plane systolic excursion.

Digital data were analyzed offline (EchoPAC 7 Workstation version 201, GE Healthcare; TomTec 4D RV Function 2.0). Measurements using 4D auto-RVQ were acquired using the 4-V matrix-array transducer on the cardiac ultrasound GE system. Images were acquired using single beats with frame rates of ≥12 FPS. Apical RV-focused four-chamber views were acquired with patients in the lateral decubitus position. The transducer position was modified for optimal simultaneous visualization of the tricuspid valve, cardiac apex, and RV outflow tract. We visualized left ventricular apex, mid–mitral valve, aortic annulus, RV apex, mid–tricuspid valve, and RV diameter in the short-axis view (Figure 1) and performed an automatic calculation to determine 4D end-diastolic volume (4D-EDV), 4D end-systolic volume (4D-ESV), 4D stroke volume (4D-SV), and 4D-EF. The longitudinal strain of RV septum and free wall and TAPSE, RV diameter (middle/basal/longitudinal RV diameter). All the measurements were performed by trained technicians blinded to clinical data, according to the guidelines of the American Society of Echocardiography (10).

### Right Heart Catheterization

Hemodynamic parameters, including mean right atrial pressure, mean PAP, and mixed venous oxygen saturation, were recorded by RHC. CO was measured using the thermodilution method. RV-CI was calculated as CO ÷ body surface area. PVR was calculated as (mean PAP – PAWP) ÷ CO [PAWP (pulmonary artery wedge pressure)]. CI <2.5 L/min/m^2^ was defined as reduced right heart function (7). The interval between echocardiography and RHC was <24 h in 76 of 103 participants. For the other 27 patients, RHC was performed >24 h after echocardiography; therefore, their hemodynamic indices were not included in the analysis.

### Statistical Analysis

Analyses were performed using SPSS version 20.0 (SPSS, Inc., Chicago, IL, USA) and MedCalc 19.0.5. Continuous data are presented as mean ± standard deviation. Categorical data are presented as absolute numbers or percentages. Differences between groups were analyzed using the χ^2^ test. Pearson correlation analysis was performed to evaluate the relationship between 4D auto-RVQ and strain, as well as those parameters measured using the reference method. Interobserver agreement for qualitative analysis score using 4D auto-RVQ manual and software assessments was calculated using the intraclass correlation coefficient. Receiver operating characteristic (ROC) curves were used to investigate and compare the predictive ability of 4D auto-RVQ parameters to evaluate right heart function. A *p*-value of <0.05 was considered statistically significant.

## Results

There were 103 adult patients with PHTN and 25 healthy controls from September 2017 to December 2018. We excluded 27 patients on whom RHC was not performed on the same day as echocardiography. Finally, 76 patients diagnosed with PHTN by RHC were recruited. We divided patients with PHTN (*n* = 76) into three groups according to echocardiographic image quality as follows: high image quality (*n* = 24), average image quality (*n* = 48), and poor image quality (*n* = 4). Clinical and conventional echocardiographic and 4D auto-RVQ characteristics of adult patients with PHTN are described in Table 1.

**Table 1:** Clinical, 2DE, and 4D auto-RVQ characteristics of 76 patients with pulmonary arterial hypertension.

**Variables**	**PHTN (*n* = 76)**	**Healthy control (*n* = 25)**	***p*****-value**
Age (years)	37.13 ± 13.46	36.28 ± 12.56	>0.05
Gender (male)	24 (31.6%)	9 (21.4%)	>0.05
BSA (m^2^)	1.57 ± 0.26		
NT-proBNP (pg/mL)	932.85 (322.2, 2,155.5)		
**Hemodynamics (*****n*** **= 76)**			
RAP (mm Hg)	4.56 ± 4.01		
mPAP (mm Hg)	53.22 ± 14.58		
RV-CI (L/min/m^2^)	2.95 (2.38, 3.50)		
PVR (dyn·s·cm^−5^)	940.29 (771.29, 1,236.50)		
SvO_2_ (%)	69.39 ± 5.75		
6 MWD (m)	404.82 ± 98.74		
**Clinical classification**			
IPAH	40 (52.6%)		
CTEPHTN	15 (19.7%)		
CTD-PAH	10 (13.2.0%)		
PAH after operation of CHD	5 (6.6%)		
**2DE RV characteristics**			
RVD (mm)	30.0 (25.0, 36.0)	22.0 (19.3, 24.0)	<0.001
RAD (mm)	42.0 (36.0, 53.0)	33.0 (29.0, 37.5)	<0.001
RV-FAC	31.88 ± 1.22	48.74 ± 9.83	0.001
TAPSE (mm)	16.0 (15.0, 20.0)	22.0 (19.8, 24.0)	<0.001
TR (m/s)	4.3 (3.6, 4.7)	1.86 (0.75, 2.28)	<0.001
TV E/A	1.2 (0.7, 1.5)	1.29 (1.12, 1.41)	0.081
LVEF (%)	69.14 ± 7.33	68.56 ± 6.38	0.769
**4D auto-RVQ characteristics**			
RV-ESV (mL)	109.57 ± 47.51	54.70 ± 20.73	<0.001
RV-SV (mL)	34.62 ± 14.00	41.77 ± 13.37	0.047
RV-EF (%)	24.99 ± 7.86	43.69 ± 7.23	<0.001
RVLS (s, %)	−7.36 ± 5.10	−12.36 ± 3.83	0.0001
RVLS (fw, %)	−11.27 ± 5.66	−20.51 ± 6.45	<0.001
RVD (basal, mm)	39.08 ± 6.20	27.77 ± 4.09	<0.001
RVD (mid, mm)	47.70 ± 10.22	35.15 ± 15.58	<0.001
RVD (long, mm)	71.10 ± 12.10	64.57 ± 11.12	0.035
TAPSE (mm)	10.60 ± 3.93	17.75 ± 1.94	<0.001
RV-FAC (%)	19.42 ± 6.23	37.82 ± 9.35	<0.001

*Continuous variables are described as mean ± standard deviation if they are normally distributed, whereas those with a skewed distribution are described as median (interquartile range). Categorical data are described as counts (proportions). PAH, pulmonary arterial hypertension; BSA, body surface area; WHOFC, World Health Organization functional class; NT-proBNP, N-terminal pro-brain natriuretic peptide; RAP, right atrial pressure; mPAP, mean pulmonary arterial pressure; RV-CI, right ventricular cardiac index; PVR, pulmonary vascular resistance; SvO_2_, mixed venous oxygen saturation; 6MWD, 6-min walking distance; IPAH, idiopathic PAH; CTEPHTN, chronic thromboembolic pulmonary hypertension; CTD-PAH, connective tissue disease-induced PAH; CHD, congenital heart disease; 2DE, two-dimensional echocardiography; RV, right ventricular; RA, right atrial; D, diameter; RVAW, right ventricular anterior wall; TAPSE tricuspid annular plane systolic excursion; TR, tricuspid regurgitation velocity; TV E/A, tricuspid valve blood early and atrium flow; LVEF, left ventricle ejection fraction; RVLS (s, %), right ventricular septum longitudinal strain; RVLS (fw, %), right ventricular free wall longitudinal strain; TAPSE, tricuspid annular plane systolic excursion; ESV, end-systolic volume; FAC, fractional area change; RVD (basal/mid/long), right ventricular diameter (basal, middle, longitudinal)*.

There were no significant differences in age and sex between the PHTN and control groups. Patients with PHTN were predominantly in World Health Organization functional class II or III, with a markedly decreased 6-min walking distance, increased mean PAP, decreased CO, and increased PVR.

The intraclass correlation coefficient is listed in Table 2. Interobserver variability showed significant agreement with a 4D auto-RVQ method for most of the measurements except 4D-EDV.

**Table 2:** Interobserver variability with 4D auto-RVQ in PHTN and Healthy controls.

	**Intraclass correlation**	**95% CI (lower)**	**95% CI (upper)**	***p*****-value**
RV-EDV (mL)	0.012	−0.167	0.190	0.447
RV-ESV (mL)	0.840	0.778	0.886	< 0.001
RV-SV (mL)	0.480	0.330	0.607	< 0.001
RV-EF (%)	0.749	0.659	0.819	< 0.001
RVLS (s, %)	0.261	0.087	0.420	0.002
RVLS (fw, %)	0.317	0.147	0.469	< 0.001
RVD (basal, mm)	0.508	0.271	0.668	0.002
RVD (mid, mm)	0.653	0.537	0.745	< 0.001
RVD (long, mm)	0.541	0.400	0.656	< 0.001
TAPSE (mm)	0.182	0.003	0.350	0.023
RV-FAC (%)	0.631	0.508	0.728	< 0.001

*RV, right ventricular; EDV, end-diastolic volume; ESV, end-systolic volume; EF, ejection fraction; RVLS (s, %), right ventricular septum longitudinal strain; RVLS (fw, %), right ventricular free wall longitudinal strain; TAPSE, tricuspid annular plane systolic excursion; FAC, fractional area change; RVD (basal/mid/long), right ventricular diameter (basal, middle, longitudinal)*.

We performed a correlation analysis of the 4D auto-RVQ indices and RHC indices in all PHTN cases (*n* = 76). Table 3 shows that there was no significant correlation between the RHC and most of the 4D indices in all PHTN patients.

**Table 3:** Correlation between RHC and 4D auto-RVQ indices in all PHTN cases (*n* = 76).

	**RV-CI (L/min/m^2^)**		**mRAP (mm Hg)**		**mPAP (mm Hg)**		**PVR (dyn·s·cm^–5^)**	
**4D indices**	**R**	**P**	**R**	**P**	**R**	**P**	**R**	**P**
RV-EDV (mL)	−0.131	0.268	0.221	0.061	0.223	0.056	0.080	0.496
RV-ESV (mL)	−0.153	0.196	0.225	0.055	0.269	0.020	0.124	0.294
RV-SV (mL)	−0.030	0.801	0.162	0.170	0.051	0.664	−0.045	0.700
RV-EF (%)	0.162	0.171	−0.135	0.257	−0.24	0.035	−0.195	0.096
RVLS (s, %)	0.037	0.758	−0.250	0.033	0.143	0.223	0.101	0.392
RVLS (fw, %)	−0.095	0.425	−0.126	0.289	0.140	0.235	0.168	0.152
RVD (basal, mm)	−0.266	0.031	0.275	0.025	0.081	0.513	0.063	0.612
RVD (mid, mm)	−0.073	0.541	0.103	0.388	−0.01	-0.078	0.030	0.802
RVD (long, mm)	0.029	0.806	0.182	0.126	0.008	0.948	−0.164	0.166
TAPSE (mm)	0.226	0.056	0.072	0.546	−0.078	0.511	−0.268	0.022
RV-FAC (%)	0.152	0.203	−0.099	0.406	−0.165	0.162	−0.203	0.085

*RV, right ventricular; EDV, end-diastolic volume; ESV, end-systolic volume; EF, ejection fraction; RVLS (s, %), right ventricular septum longitudinal strain; RVLS (fw, %), right ventricular free wall longitudinal strain; TAPSE, tricuspid annular plane systolic excursion; FAC, fractional area change; RVD (basal/mid/long), right ventricular diameter (basal, middle, longitudinal); RV-CI, right ventricular cardiac index; mRAP, mean right atrial pressure; mPAP, mean pulmonary arterial pressure; PVR, pulmonary vascular resistance*.

In 24 patients with high image quality, 4D auto-RVQ indices had a very good correlation with RV-CI, and 4D-ESV had a good correlation with all important RHC indices (Table 4).

**Table 4:** Correlation between RHC and 4D auto-RVQ indices in the high-quality image PHTN group (*n* = 24).

	**RV-CI (L/min/m^2^)**		**mRAP (mm Hg)**		**mPAP (mm Hg)**		**PVR (dyn·s·cm^–5^)**	
**4D indices**	**R**	**P**	**R**	**P**	**R**	**P**	**R**	**P**
RV-EDV (mL)	−0.579	0.007	0.548	0.012	0.503	0.024	0.444	0.050
RV-ESV (mL)	−0.642	0.002	0.529	0.016	0.501	0.024	0.476	0.034
RV-SV (mL)	−0.126	0.596	0.358	0.122	0.281	0.230	0.145	0.541
RV-EF (%)	0.529	0.016	−0.211	0.371	−0.169	0.477	−0.293	0.211
RVLS (s, %)	−0.172	0.469	−0.225	0.340	0.351	0.129	0.565	0.009
RVLS (fw, %)	−0.519	0.019	−0.032	0.893	0.120	0.615	0.506	0.023
RVD (basal, mm)	−0.691	0.001	0.435	0.063	0.621	0.005	0.606	0.006
RVD (mid, mm)	−0.501	0.025	0.312	0.180	0.290	0.229	0.641	0.002
RVD (long, mm)	−0.204	0.402	0.678	0.001	0.720	0.0001	−0.056	0.819
TAPSE (mm)	0.070	0.775	0.357	0.133	−0.038	0.879	−0.324	0.176
RV-FAC (%)	0.510	0.026	−0.109	0.657	−0.163	0.504	−0.372	0.117

*RV, right ventricular; EDV, end-diastolic volume; ESV, end-systolic volume; EF, ejection fraction; RVLS (s, %), right ventricular septum longitudinal strain; RVLS (fw, %), right ventricular free wall longitudinal strain; TAPSE, tricuspid annular plane systolic excursion; FAC, fractional area change; RVD (basal/mid/long), right ventricular diameter (basal, middle, longitudinal); RV-CI, right ventricular cardiac index; mRAP, mean right atrial pressure; mPAP, mean pulmonary arterial pressure; PVR, pulmonary vascular resistance*.

When all PHTN patients with high, average, and poor image quality data combined, the 4D auto-RVQ indices were not clinically meaningful based on the ROC curve analysis (Table 5).

**Table 5:** All PHTN cases (*n* = 76) of 4D auto-RVQ areas of receiver operating characteristic (ROC) curves of various right ventricular function parameters.

	**ROC curve area**	**95% CI**	***p*****-value**	**Cutoff**	**Sensitivity (%)**	**Specificity (%)**	**Youden index**
RV-EDV (mL)	0.651	0.522–0.766	0.070	> 153.30	66.67	76.74	0.434
RV-ESV (mL)	0.664	0.535–0.777	0.040	> 117.50	66.67	79.07	0.457
RV-SV (mL)	0.561	0.431–0.685	0.485	> 40.20	42.86	81.40	0.242
RV-EF (%)	0.580	0.450–0.703	0.270	≤25.63	76.19	51.16	0.273
RVLS (s, %)	0.525	0.396–0.651	0.770	> −9.39	71.43	11.63	0.169
RVLS (fw, %)	0.646	0.516–0.761	0.070	> −9.78	61.90	72.09	0.340
RVD (basal, mm)	0.643	0.505–0.766	0.080	> 39.4	70.59	57.50	0.281
RVD (mid, mm)	0.585	0.455–0.707	0.320	> 55.20	42.86	86.05	0.289
RVD (long, mm)	0.566	0.435–0.690	0.440	> 76.4	42.86	85.71	0.286
TAPSE (mm)	0.618	0.487–0.738	0.140	≤6.5	33.33	90.48	0.238
RV-FAC (%)	0.658	0.527–0.773	0.029	≤19.17	71.43	64.29	0.357

*RV, right ventricular; EDV, end-diastolic volume; ESV, end-systolic volume; EF, ejection fraction; RVLS (s, %), right ventricular septum longitudinal strain; RVLS (fw, %), right ventricular free wall longitudinal strain; TAPSE, tricuspid annular plane systolic excursion; FAC, fractional area change; RVD (basal/mid/long), right ventricular diameter (basal, middle, longitudinal)*.

**Figure 2:**
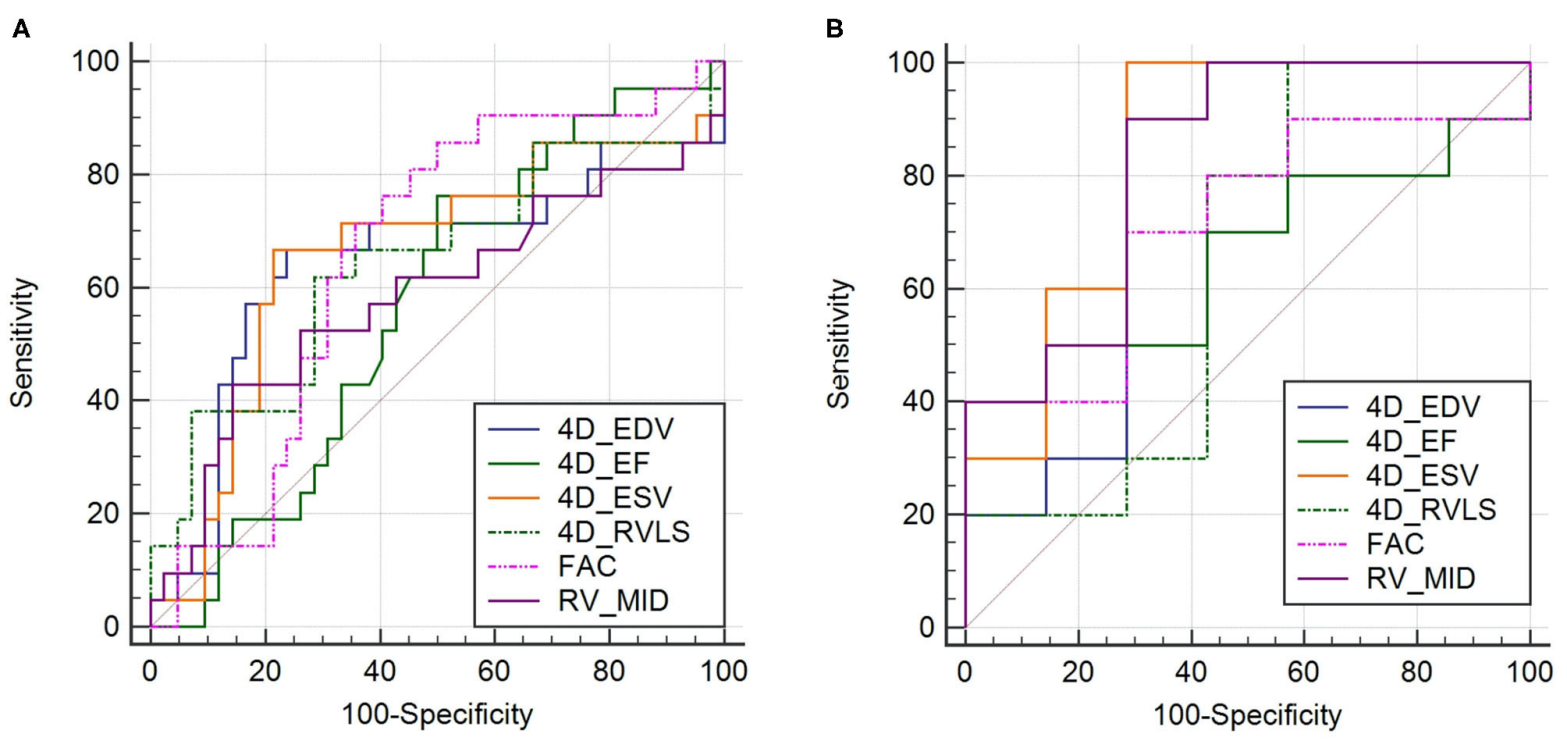
Receiver operating characteristic curves in all patient groups of PHTN (*n* = 76) of 4D auto-RVQ areas of **(A)** and in the high-quality image group of PHTN (*n* = 24) **(B)**. 4D_EDV, four-dimensional end-diastolic volume; 4D_ESV, four-dimensional end-systolic volume; 4D_EF, four-dimensional ejection fraction; 4D-RVLS, right ventricular longitudinal strain of the free wall; FAC, fractional area change; RV_MID, mid–right ventricular diameter.

ROC curves (Figure 2, Table 6) illustrated that all six 4D auto-RVQ parameters could predict right heart function in the subgroup of high-quality images, especially mid-RV diameter [area under the curve (AUC) 0.935; 95% confidence interval (CI), 0.714–0.997; *p* < 0.001]. A mid-RV diameter of >50.8 mm had an 85.7% sensitivity and a 90.9% specificity. A RV-ESV of >121.5 mL had a 71.4% sensitivity and a 100% specificity, as well as a Youden index of 0.714 to predict RV-CI (AUC, 0.890; 95% CI, 0.654–0.986; *p* < 0.001).

**Table 6:** High-quality image group of PHTNs (*n* = 24) of 4D auto-RVQ areas of receiver operating characteristic (ROC) curves of various right ventricular function parameters.

	**ROC curve area**	**95% CI**	***p*****-value**	**Cutoff**	**Sensitivity (%)**	**Specificity (%)**	**Youden index**
RV-EDV (mL)	0.831	0.583–0.963	0.007	> 153.94	71.43	100	0.714
RV-ESV (mL)	0.890	0.654–0.986	0.001	> 121.50	71.43	100	0.714
RV-SV (mL)	0.636	0.381–0.845	0.421	> 41.45	57.14	90.91	0.481
RV-EF (%)	0.779	0.525–0.937	0.002	≤26.21	100	54.55	0.545
RVLS (s, %)	0.506	0.266–0.745	0.960	> −5.73	42.86	81.82	0.245
RVLS (fw, %)	0.773	0.518–0.993	0.019	> −9.95	71.43	81.82	0.532
RVD (basal, mm)	0.831	0.583–0.963	0.001	> 34.15	85.71	81.82	0.675
RVD (mid, mm)	0.935	0.714–0.997	0.000	> 50.8	85.71	90.91	0.766
RVD (long, mm)	0.600	0.340–0.824	0.540	> 78.6	42.86	100	0.428
TAPSE (mm)	0.557	0.302–0.792	0.714	≤11.3	71.43	60	0.314
RV-FAC (%)	0.800	0.539–0.951	0.011	≤14.66	71.43	90	0.614

*RV, right ventricular; EDV, end-diastolic volume; ESV, end-systolic volume; EF, ejection fraction; RVLS (s, %), right ventricular septum longitudinal strain; RVLS (fw, %), right ventricular free wall longitudinal strain; TAPSE, tricuspid annular plane systolic excursion; FAC, fractional area change; RVD (basal/mid/long), right ventricular diameter (basal, middle, longitudinal)*.

## Discussion

Despite its clinical importance, the study of RV function is technically challenging, in patients with PHTN. It is usually very difficult to evaluate RV morphology and function using 2DE. Recently, 3DE and 4DE have been introduced and are reportedly feasible and clinically applicable for RV volumetric quantification in patients with acquired RV pressure or volume overload (11). At present, the advantages of 4DE could better reflect RV size and function. As a new technology, 4D auto-RVQ can combine the 3D echocardiographic morphological, functional, and strain characteristics for comprehensive RV evaluation. It has the potential to reflect changes in RV function and pulmonary artery hemodynamics (9, 12).

Our results showed that 4D auto-RVQ, like 3D echocardiography, can accurately detect RV volume measurements in both PHTN patients and healthy control with good interobserver reproducibility (13, 14). However, the intraclass correlation coefficient of 4D-EDV was suboptimal. This may be due to the large RV volume in patients with PHTN so the right ventricle could not be completely included in some cases. Furthermore, 4D auto-RVQ can possibly detect differences in RV diameter, volume, and function. RV volume and basal and middle diameters were significantly enlarged in PHTN, but the longitudinal diameter was not significantly changed. This may be due to lateral expansion of the RV in patients with PHTN. In terms of the strain indices measured by 4D auto-RVQ, the longitudinal strains of the septum and free wall were significantly lower in patients with PHTN compared with the control group (15, 16). Similar to our findings, some reports found that the longitudinal strain of the septum and free wall had the potential to independently predict intermediate- to high-risk features in PAH patients (17). Smith also showed that RV longitudinal strain of the free wall (hazard ratio, 7.63; 95% CI, 1.76–10.27; *p* < 0.001) was a significant determinant of all-cause mortality (18).

When analyzing the data from RHC and 4D auto-RVQ indices, we observed a good correlation between 4D quantification and RHC in patients with high-quality images, but not in the other two groups with suboptimal imaging quality. RV-ESV by 4D auto-RVQ tracked well with RV-CI, mean right atrial pressure, mean PAP, and PVR measurements with RHC. We propose that RV-ESV may be an effective indicator of right heart function and hemodynamic changes in patients with PHTN. RV function analysis needs to take into consideration of its afterload including PVR. Measurement of 4D-ESV, 4D-RV strain, and RV basal and middle diameters had a good correlation with PVR. In the high-quality image group, all six 4DE parameters predicted right heart function. The indices with the best predictive power were RV middle diameter and 4D-ESV. We did notice that some authors previously used RV-EF as an index compared with cardiac magnetic resonance imaging (19, 20).

In summary, 4D auto-RVQ is a possibly useful quantitative tool to measure RV function with validation by RHC and provide meaningful data reflecting RV function. It is important to obtain high-quality RV images and to include the entire RV within the scanning sector. Previous studies have shown that in patients with chronic PHTN, 3D and 3D speckle-tracking echocardiography parameters for global and regional RV dysfunction were better than conventional echocardiographic indices (21, 22). The software for 4D auto-RVQ generates functional parameters from 4D datasets, making it an easy tool to implement in clinical practice and reducing the amount of time spent using multiple software parameters to simultaneously analyze 4D datasets and speckle-tracking echocardiography.

RHC remains the gold standard for RV hemodynamic evaluation, but 4D auto-RVQ has the potential to be included in routine clinical applications because it provides a relatively quantitative evaluation method for RV function in PHTN patients if high-quality echocardiographic images were acquired.

## Limitations

There are several limitations to our data and results. As a single-center study in a cardiac referral center, we might have had selection bias. Among 103 patients, 27 patients did not simultaneously undergo ultrasonography and RHC and were excluded from the final analysis. World Health Organization functional class IV patients were not enrolled in the study. Our data are mainly applicable to patients with high-quality echocardiographic images. Our sample size was relatively small for an ROC analysis. Finally, we collected only the RHC data of PHTN without the results of cardiac magnetic resonance imaging. Thus, it is difficult to evaluate changes in cardiac volume in real time.

## Conclusion

In conclusion, 4D auto-RVQ is a new method to estimate RV function and hemodynamic changes compared with gold-standard RHC in patients with PHTN only if high-quality echocardiographic images were acquired.

## Data Availability Statement

The raw data supporting the conclusions of this article will be made available by the authors, without undue reservation.

## Ethics Statement

The studies involving human participants were reviewed and approved by the Ethics Committee of Fuwai Hospital (No. 2018-1063). Written informed consent to participate in this study was provided by the participants' legal guardian/next of kin.

## Author Contributions

WW, BL, and MH drafted the manuscript. LN, YT, JL, JW, SY, and HL contributed to data collection and statistical analysis. HW, DH, CX, and ZZ provided supervision and revised the manuscript. CX contributed to the conception, design, and supervision of the study. All authors contributed to the article and approved the submitted version.

## Conflict of Interest

The authors declare that the research was conducted in the absence of any commercial or financial relationships that could be construed as a potential conflict of interest.
